# S100A8 and S100A9 Positive Cells in Colorectal Carcinoma: Clinicopathological Analysis

**DOI:** 10.1155/2014/943175

**Published:** 2014-10-13

**Authors:** Cumhur İbrahim Başsorgun, Betül Ünal, Nuray Erin, Anıl Özlük, Özlem Ceren Uzun, Gülsüm Özlem Elpek

**Affiliations:** ^1^Department of Pathology, Faculty of Medicine, Akdeniz University, 07070 Antalya, Turkey; ^2^Department of Internal Medicine, Faculty of Medicine, Akdeniz University, 07070 Antalya, Turkey

## Abstract

*Introduction*. In colorectal carcinoma, tumoral tissues infiltrate with various immune/inflammatory cells along their invasive margins and the increased S100A8/A9 expression in these immune cells infiltrating the tumor has recently been demonstrated. We examined S100A8/A9 as a potential therapeutic target in the treatment of colorectal carcinoma. *Materials and Methods*. The current study included a sample of 80 patients diagnosed with CRC (30 cases with distant metastasis, 30 cases with lymph node metastasis, and 20 cases with no metastasis). Peritumoral and intratumoral S100A8 and S100A9 expressing inflammatory cells were counted in primary tumors and their metastasis and correlated with clinicopathological parameters. *Results*. The peritumoral and intratumoral S100A8/A9 positive cells showed no correlation with age, gender, or depth of tumor invasion. However higher counts of peritumoral and intratumoral S100A8/A9 positive cells were associated with larger tumor size, higher grade, and the presence of metastasis (*P* < 0.05). *Conclusion*. Our study also found significantly higher number of S100A8/A9 positive cells in the tumor microenvironment among patients with large tumor size, high grade, and metastatic disease. Moreover, in our study, we observed that the expression in the tumor metastasis appeared similar to that of primary tumor.

## 1. Introduction

S100A8 (calgranulin A or migration inhibitory factor-related protein-8 (MRP-8)) and its binding partner S100A9 (calgranulin B or MRP-14) are two members of a multigenic family of cytoplasmic Ca2+-binding proteins [1]. These proteins are synthesized by the myeloid cell group mainly composed of neutrophils, monocytes, immature myeloid cells, and myeloid derived suppressor cells (MDSCs) [2, 3]. Both proteins are often coexpressed, composing the heterodimeric complex of S100A8/A9 usually known as “calprotectin” [2]. Increased S100A8/A9 expression has been reported to occur in several chronic inflammatory diseases such as rheumatoid arthritis, multiple sclerosis, cystic fibrosis, tuberculosis, inflammatory bowel disease, psoriasis, and transplant rejection [4–6]. In addition to inflammatory conditions, overexpression of S100A8/A9 has also been observed in various types of cancer including stomach, prostate, breast, skin, pancreas, liver, lung, bladder, colon, and esophageal cancers [7–11].

In colorectal carcinoma, tumoral tissues infiltrate with various immune/inflammatory cells along their invasive margins, and the increased S100A8/A9 expression in these immune cells infiltrating the tumor has recently been demonstrated by certain studies. In addition, several studies have suggested that increased S100A8/A9 expression in colorectal carcinoma may play a role in tumor progression and may be associated with metastasis and histological grade. Although a few studies imply a possible relationship with tumor progression and metastasis, there is no clear correlation between increased expression of S100A8/A9 in colorectal cancer and clinicopathological parameters such as tumor progression, tumor stage, lymph node metastases, or distant metastases. The studies conducted on this topic so far have used different counting methods in assessing the S100A8/A9 expression. Research on the subject has been mostly restricted to limited comparisons of peritumoral inflammatory cells in primary tumors or intratumoral inflammatory cells and tumor cells. However, no research has been found to examine the expression in tumor metastasis and severity or its comparison with the primary tumors.

In our study, we investigated increased expression of S100A8/A9 in the tumor microenvironment in colorectal carcinoma as well as previously shown inflammatory process functions of S100A8/A9, which exhibits concentration-dependent function and forms a heterodimeric complex. We also examined S100A8/A9 as a potential therapeutic target in the treatment of colorectal carcinoma by analyzing the relationship between S100A8/A9, secreted by immune cells infiltrating the tumor along its invasive margin, and various clinicopathological parameters including tumor progression and metastasis.

## 2. Materials and Methods

The current study included a sample of 80 patients diagnosed with CRC (30 cases with distant metastasis, 30 cases with lymph node metastasis, and 20 cases with no metastasis) in the Department of Pathology, Akdeniz University Medical Faculty. The patients were surgically treated at the Department of Surgery between 2007 and 2012. The clinicopathological characteristics of the patients are summarized in Tables 1 and 2.

The median age of the 39 men was 69.6 years (range: 49–78 years) at the time of operation and that of the 41 women was 60.4 years (range: 41–84 years).

Four-micrometer-thick haematoxylin and eosin stained tissue sections from the surgical specimens fixed in 10% formalin and embedded in paraffin were reviewed and representative tissue blocks were selected. Slides were immunostained with anti-S100A8 and anti-S100A9 (1 : 50 dilution, Santa Cruz, USA) monoclonal antibodies by the avidin-biotin immunoperoxidase technique. Peritumoral and intratumoral S100A8 and S100A9 expressing inflammatory cells were counted in primary tumors and their metastasis. In each case, positive cells were counted at ×400 magnification in 10 systematically selected fields of vision and their mean number was obtained by averaging the number of positive cells per square millimeter of tissue. The research data were analyzed using SPSS 10.0. Continuous variables were compared using the Student's* t*-test, and Chi-square test was used for univariate analysis of categorical data. Tests were considered significant when their *P* values were <0.05.

## 3. Results

**Figure 1:**
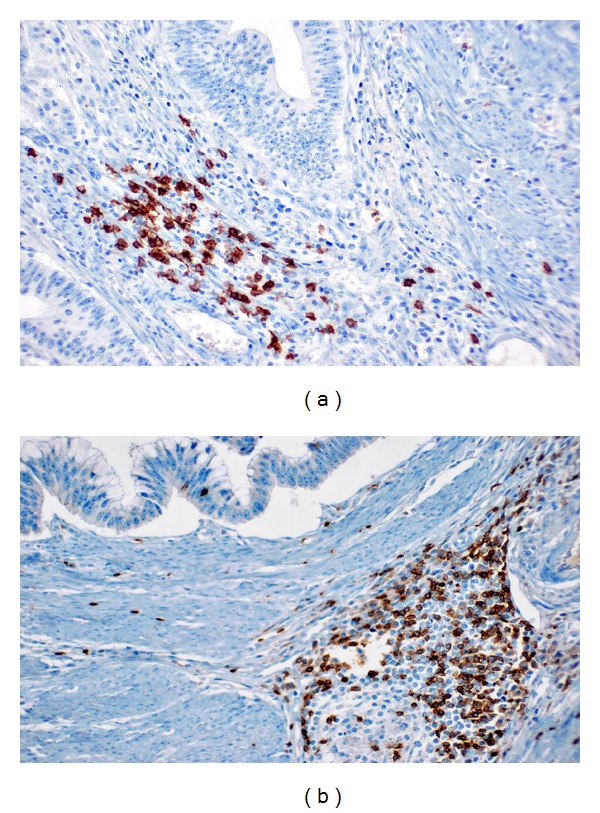
S100A8 staining in intratumoral (a) and peritumoral (b) inflammatory cells (×200).

**Figure 2:**
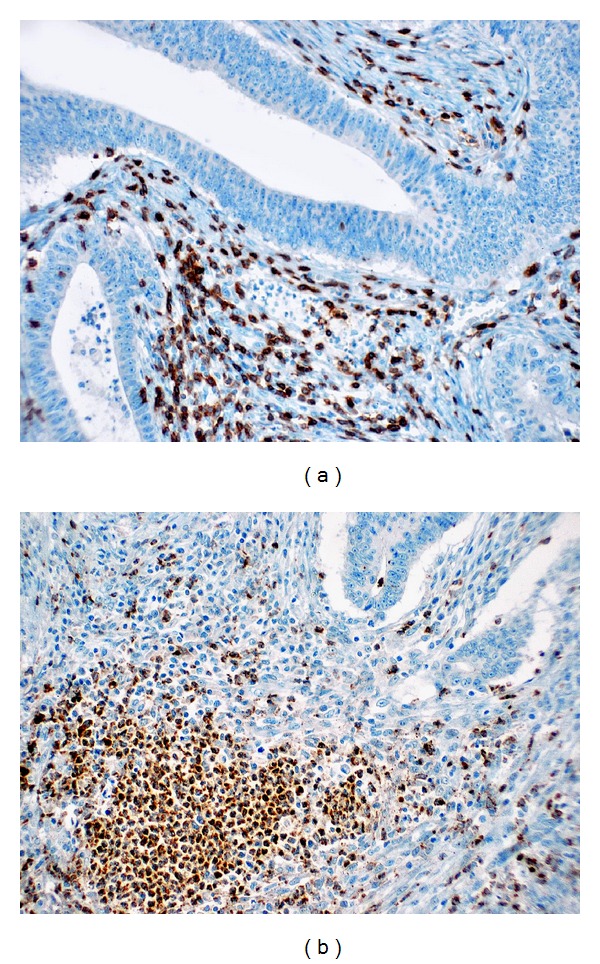
S100A9 staining in intratumoral (a) and peritumoral (b) inflammatory cells (×200).

Tumors generally contained higher number of peritumoral and intratumoral S100A9 positive cells than peritumoral and intratumoral S100A8 positive cells (Figures 1 and 2). The mean number of peritumoral and intratumoral S100A8/A9 positive cells was significantly higher in tumors with larger size, higher grade, and metastasis (*P* < 0.05) (Table 1). When patients were categorized as having median S100A8/A9 cell counts, for further statistical evaluation, the peritumoral and intratumoral S100A8/A9 positive cells showed no correlation with age, gender, or depth of tumor invasion (Table 2). However higher counts of peritumoral and intratumoral S100A8/A9 positive cells were associated with larger tumor size, higher grade, and the presence of metastasis (*P* < 0.05).

## 4. Discussion

Increased expression of S100A8/A9 in colorectal carcinoma was first shown by Stulík et al. using two-dimensional gel electrophoresis [12]. S100A8/A9 has both intracellular and extracellular functions. In the intracellular space, S100A8/A9 detects calcium, activates NADPH oxidase, and conducts arachidonic acid transport into cells [13, 14], while in the extracellular space it exhibits concentration-dependent functions. At high concentrations (>80 microgram/mL), it displays apoptotic effect on tumor cells, whereas at lower concentrations (<25 microgram/mL) it regulates the viability and migration of tumor cells, endothelial cells, and inflammatory cells and supports tumor cell growth [15–17]. MDSC plays an important part in the suppression of T cell-mediated immune response, increasing in number in inflammation and tumors. As a result of interaction with S100A8/A9 binding sites on MDSC, S100A8/A9 activates MDSC migration [18]. In other words, S100A8/A9 provides MDSC accumulation through an autocrine feedback effect. S100A8/A9 performs these effects through certain cell surface receptors. These are TLR4 and RAGE receptors, known to play a role in infection, autoimmunity, and cancer [19].

Wnt/beta-catenin pathway has a crucial role in the development of colorectal carcinoma [20].

In fact, Duan et al. demonstrated that S100A8/A9 contributed survival and migration of colorectal carcinoma cells through Wnt/beta-catenin pathway, emphasizing that it might be a potential therapeutic target in the treatment of colorectal carcinoma [19].

Our study found that S100A8/A9 positive cell count observed in the tumor microenvironment (peritumoral, intratumoral) was significantly high in the study sample of 80 patients diagnosed with colorectal adenocarcinoma (30 patients with distant metastases, 30 patients with lymph node metastasis, and 20 cases with no metastasis), and comparison with clinicopathological parameters revealed that increased number of S100A8/A9 positive cells was associated with tumor size, high grade, and metastasis. On the other hand, it was found that the number of peritumoral and intratumoral S100A8/A9 positive cells had no correlation with age, gender, and tumor invasion depth. Besides, comparison of positive cell count in the metastatic tumor tissue with positive cell count in the primary tumor did not show a statistically significant difference. This also accords with earlier observations by Duan et al., who found that S100A8/A9 expression in tumor microenvironment of colorectal carcinoma was associated with tumor cell differentiation, Dukes stage, and metastasis [19]. In addition, this study emphasized that this impact of proteins on tumor progression contributed to survival and migration of tumor cells through Wnt/beta-catenin pathway. In fact, some research has reported that Wnt/beta-catenin pathway plays a critical role in the development of colorectal carcinoma [20]. Another study by Ang et al. [21] associated S100A8/A9 expression in stromal cells of colorectal carcinoma with large tumor size. Our findings seem to be consistent with the results of these previous studies investigating the relationship between the expression and clinicopathological parameters. Also, Kim et al., who conducted a study on S100A8/A9, observed increased expression of both proteins in stromal cells in colorectal carcinomas [22] and Sheikh et al. in pancreatic cancer-associated monocytes [23].

There are also several studies that associated the increased expression of S100A8/A9 with poor differentiation in tumors of breast, thyroid, and lung which exhibit glandular differentiation [10, 24, 25]. These members of the S-100 protein family can be identified as potential therapeutic targets for intervention in cancer treatment. However, far too little attention has been paid to the correlation between the expressions of these proteins in the tumor microenvironment in colorectal carcinoma and clinicopathological parameters used to predict tumor progression.

In conclusion, it has been suggested that calcium ions play a significant role in the development of colorectal carcinoma through a direct impact on proliferation and differentiation via calcium receptors [26]. For this reason, increased expression of calcium-binding proteins S100A8 and A9, members of the S-100 protein family, in the peritumoral and intratumoral spaces in colorectal carcinoma becomes important for tumor progression.

Therefore, the analyses conducted on the expression of these proteins and tumor behavior suggest that S100A8/A9 can become potential therapeutic targets in cancer treatment. So far, a limited number of studies have reported correlation between increased expression of S100A8/A9 and parameters such as tumor differentiation, metastasis, tumor size, and Dukes stage. Consistent with this finding, our study also found significantly higher number of S100A8/A9 positive cells in the tumor microenvironment among patients with large tumor size, high grade, and metastatic disease. Moreover, in our study, we observed that the expression in the tumor metastasis appeared similar to that of primary tumor. However, further studies with larger sample sizes are warranted to substantiate the results of this study and to better understand the functions of the S100A8/A9 positive immune cells observed in the tumor microenvironment of colorectal cancer in tumorigenesis and tumor progression.

## Figures and Tables

**Table 1:** Distributions of mean ± SD and median values of S100A8/A9 positive cells in both intratumoral and peritumoral areas, *P < 0.05.

Parameters	S100A8 (intratumoral)		S100A8 (peritumoral)		S100A9 (intratumoral)		S100A8 (peritumoral)	
	Mean ± SD	Median	Mean ± SD	Median	Mean ± SD	Median	Mean ± SD	Median
Age (years)								
<64	138.06 ± 82.3	83	213.04 ± 97.21	166	161.68 ± 92.04	135	338.73 ± 144.2	270
>64	140.96 ± 73.5	96	227.98 ± 112.4	191	173.78 ± 90.2	154	323.87 ± 127.79	250
Gender								
Female	149.66 ± 87.2	88	222.81 ± 112.4	157	158.74 ± 69.32	136	325.63 ± 172.5	230
Male	129.28 ± 59.2	97	218.21 ± 89.12	176	176.72 ± 81.54	170	336.97 ± 148.11	269
Size (cm)∗								
<5.7	105.9 ± 52.3	74	154.77 ± 67.31	149	118.82 ± 57.91	110	285.46 ± 133.6	262
>5.7	173.56 ± 76.02	130	286.25 ± 133.14	181	216.64 ± 109.53	157	377.14 ± 144.51	315
Grade∗								
Low	108.75 ± 42.5	62	168.53 ± 67.71	132	130.79 ± 63.42	119	295.14 ± 121.33	203
High	170.71 ± 78.01	136	272.49 ± 114.99	143	204.67 ± 75.33	186	367.46 ± 136.89	332
Depth of invasion								
T1	137.85 ± 46.59	72	220.51 ± 67.86	132	162.3 ± 59.32	119	329.18 ± 112.23	212
T2	132.98 ± 58.98	107	203.95 ± 102.53	183	167.73 ± 68.15	129	325.12 ± 107.54	284
T3	149.36 ± 73.76	124	223.86 ± 106.12	271	169.2 ± 47.32	190	333.25 ± 101.04	252
T4	138.73 ± 75.63	123	233.72 ± 98.21	198.	171.69 ± 76.02	147	337.65 ± 144.51	217
Lymph nodes∗								
N0	110.98 ± 46.34	62	171.85 ± 87.86	132	104.51 ± 95.91	68	273.45 ± 128.43	201
N1	129.16 ± 54.97	107	208.32 ± 102.53	183	169.04 ± 68.5	129	305.68 ± 103.54	264
N2	179.05 ± 43.76	114	281.86 ± 106.2	212	229.64 ± 98.89	108.	414.77 ± 101.04	371
Distant metastasis∗								
Absent	114.51 ± 28.2	45	182,27 ± 3.12	82	140.21 ± 47.21	69	182.3 ± 77.31	146
Present	164.95 ± 73.5	122	258.75 ± 123.1	181	195.25 ± 94.24	173	480.3 ± 144.51	320

**Table 2:** Distribution of clinicopathologic parameters according to median values S100A8/A9 positive cells in both intratumoral and peritumoral areas. (Chi-square test, *P < 0.05.)

Parameters	S100 A8 (intratumoral)			S100 A8 (peritumoral)			S100 A9 (intratumoral)			S100 A9 (peritumoral)		
	<123 (%)	>123 (%)	P*	<198 (%)	>198 (%)	*P*	<147 (%)	>147 (%)	*P*	<297 (%)	>297 (%)	*P*
Age (years)												
<64	19 (55.9)	15 (44.1)	0.16	20 (58.8)	14 (41.2)	0.12	19 (55.9)	15 (44.1)	0.27	20 (58.8)	14 (41.2)	0.12
>64	20 (43.5)	26 (56.5)		19 (41.3)	27 (58.7)		20 (43.5)	20 (43.5)		19 (41.3)	27 (58.7)	
Gender												
Female	17 (41.5)	24 (58.5)	0.54	15 (36.6)	26 (63.4)	0.32	16 (39)	25 (61)	0.07	16 (39)	25 (61)	0.08
Male	22 (56.4)	17 (43.6)		24 (61.5)	15 (38.5)		23 (59)	16 (41)		23 (59)	16 (41)	
Size (cm)∗												
<5.7	28 (63.6)	16 (36.4)	0.003	29 (65.9)	15 (34.1)	0.001	29 (65.9)	15 (34.1)	0.001	31 (70.5)	13 (29.5)	0.001
>5.7	11 (30.6)	25 (69.4)		10 (27.8)	26 (72.2)		10 (27.8)	26 (72.2)		8 (22.2 )	28 (77.8)	
Grade∗												
Low	24 (68.6)	11 (31.4)	0.003	26 (74.3)	9 (25.7)	0.001	25 (71.4)	10 (28.6)	0.001	27 (77.1)	8 (22.9)	0.001
High	15 (33.3)	30 (66.7)		13 ( 28.9)	32 (71.1)		14 (31.1)	31 (68.9)		12 (28.9)	33 (73.3)	
Depth of invasion												
T1	17 (65.4)	9 (34.6)	0.8	16 (61.5)	10 (38.5)	0.23	17 (65.4)	9 (34.6)	0.8	14 (53.8)	12 (46.2)	0.65
T2	11 (55)	9 (45)		10 (50)	10 (50)		11 (55)	9 (45)		10 (50)	10 (50)	
T3	6 (35.3)	11 (64.7)		8 (47.1)	9 (52.9)		6 (35.3)	11 (64.7)		9 (52.9)	8 (47.1)	
T4	5 (29.4)	12 (70.6)		5 (29.4)	12 (70.6)		5 (29.4)	12 (70.6)		6 (35.3)	11 (64.7)	
Lymph nodes∗												
N0	20 (60.6)	13 (39.4)	0.001	20 (60.6)	13 (39.4)	0.01	20 (60.6)	13 (39.4)	0.003	19 (54.5)	14 (45.5)	0.01
N1	16 (64)	9 (36)		14 (56)	11 (44)		15 (60)	10 (40)		14 (56)	11 (44)	
N2	3 (13.6)	19 (86.4)		5 (22.7)	17 (77.3)		4 (18.2)	18 (81.8)		7 (31.8)	15 (68.2)	
Distant metastasis∗												
Absent	16 (80)	4 (20)	0.001	12 (80)	8 (40)	0.001	15 (75)	5 (25)	0.007	14 (70)	6 (30)	0.007
Present	23 (38.3)	37 (61.7)		23 (38.3)	37 (61.7)		24 (40)	36 (60)		24 (40)	36 (60)	
